# Supplementary material for: Spin Density Topology
Source: Molecules. 2020 Aug 2;25(15):3537. doi: 10.3390/molecules25153537 (PMC7436107; doi:10.3390/molecules25153537)
Supplement: Supplementary file 1 [file molecules-25-03537-s001.pdf]

molecules

MDPI

Supplementary information

Spin Density Topology

Giovanna Bruno [1], Giovanni Macetti [2], Leonardo Lo Presti [1] and Carlo Gatti [3,4*]

1 Dipartimento di Chimica, Università degli Studi di Milano, via Golgi 19, 20133 Milano, Italy; giovanna.bruno@unimi.it, and leonardo.lopresti@unimi.it
2 Université de Lorraine & CNRS, Laboratoire de Physique et Chimie Théoriques (LPCT), 1 Boulevard Arago, F-57078 Metz, France; giovanni.macetti@univ-lorraine.fr
3 CNR-SCITEC, Istituto di Scienze e Tecnologie Chimiche sezione di via Golgi, via Golgi 19, 20133 Milano, Italy;
4 Istituto Lombardo, Accademia di Scienze e Lettere, via Brera 28, 20100 Milano, Italy
* Correspondence: c.gatti@scitec.cnr.it

Received: date; Accepted: date; Published: date

S1. Further details about methods and computations

CASSCF(8,8) wavefunction: The Slater determinant expansion of the CASSCF (8,8) wavefunction includes 3136 configurations of the correct symmetry and spin multiplicity.

UHF wavefunction: In case one would like to repeat our analysis, it is worth reporting that in order to save the UHF spin contamination annihilated solution in the *wfn* file, the "pop" option involving natural orbitals (e.g., pop=no or pop=noab) needs to be selected. Otherwise, the standard spin-contaminated wave-function is unavoidably saved in the *wfn* file, even if the UHF spin annihilation (IOP(5/14=2)) option is activated. The *wfn* file is written by Gaussian-09 code [41] (command line, output=wfn command) and serves as a wavefunction input for the AIMPAC [42] and Multiwfn [43] program packages (see below).

Electron Spin densities: For the ROHF and UHF models, the electron-spin-density was calculated from the naturals orbitals obtained from separate diagonalizations of the α- and β-density matrices (Gausian-09, pop=noab option) and using either the modified AIMPAC or Multiwfn Packages. Both these codes read natural orbital information from the Gaussian 09 *wfn* files. As discussed in the SI of our previous Chemical Science study [19], the Gaussian09 package is unable to calculate and save spin-density information in the case of the CASSCF method, at least using a standard procedure. However, if IOP (5/72=1) and "SlaterDet" (Slater Determinants) options are activated, spin-density information is correctly passed to L6XX (XX=01,…etc) links. The SlaterDet option requires, in our case, the introduction of the "1 1" string in the penultimate row of the Gaussian 09 input file, just before the name selected for the *wfn* file. Proceeding this way, a correct α-density is obtained through the pop=noa option (yet, neither the correct spin density through pop=noab, nor the correct β-density through pop=nob are recovered). However from the total electron density and its α component, the electron spin density and electron-spin-density Laplacian are easily obtained, at any point **r**, by the differences: $s(\mathbf{r}) = 2\rho_\alpha(\mathbf{r}) - \rho(\mathbf{r})$; $\nabla^2 s(\mathbf{r}) = 2\nabla^2 \rho_\alpha(\mathbf{r}) - \nabla^2\rho(\mathbf{r})$. Hereinafter, we name as a <t –v> *wfn* file a *wfn* file composed, in sequence, by n1 naturals orbitals yielding the scalar t and n2 orbitals yielding the scalar –v. For instance, in our case, the CASSCF spin density will be correctly recovered by using a $<2\rho_\alpha - \rho>$ *wfn* file.

Multiwfn code. Evaluation of the CASSCF spin density and of spin-density basins properties: In the case of the Multiwfn package, which we used for evaluating basins bounded by s=0 surfaces and enclosing either a s_+ or a s_- region, a further special trick had to be devised for handling CASSCF spin densities. The $<2\rho_\alpha - \rho>$ *wfn* file (see above) cannot be used for evaluating the spin density through the

"basin analysis" option of the main functions list available in Multiwfn. Indeed, this code is unable to properly distinguish the α- from the β-part if the *wfn* file is written in such a form. Therefore, we first build up a <ρ -ρ_α> *wfn* file, thus containing the ρ_β information and where all orbitals having occupation numbers greater than one were duplicated and assigned an halved occupation number, resulting in an equivalent <ρ -ρ_α> *wfn* file or, more simply, in a <ρ_β> *wfn* file. Then, by combining in sequence the <ρ_α> *wfn* file and the newly generated <ρ_β> *wfn* file with reversed occupation numbers, a spin density <ρ_α -ρ_β> *wfn* file was eventually obtained. The resulting file is recognized by Multiwfn as a post-UHF .wfn file and the α and β parts can now be properly separated and handled by this code. Using the <ρ_α -ρ_β> *wfn* file, the basins recovered by the basin analysis section and by selecting the electron density as a property are those of the spin density. These basins correspond to s_+ or s_- regions, each enclosing a (3, -3) or a (3, +3) spin-density CP, respectively and the electron populations calculated by Multiwfn are, thereby, their electron spin populations. Conversely, the electron populations of such basins may be evaluated by activating the integration of the spin-density function within such basins. As a check of the correctness of our manipulations, we got a value of 1.99984 and 9.9969 e^- for the electron spin and electron populations, respectively, by summing up the corresponding population values over the seven distinct CASSCF spin-density basins. All these analyses were performed using the Multiwfn lunatic grid (0.04 Bohr grid spacing in a box of volume 20x20x20 $Bohr^3$).

Multiwfn code. Evaluation of the basin volumes: Four simple user-functions were implemented by C. Gatti to calculate the $V1_\Omega$ and $V2_\Omega$ volumes (see main text) of the Ω basins bounded by s=0 surfaces for either the ROHF/UHF wavefunctions or the CASSCF one (in this latter case, from a *wfn* file manipulated as described above).

AIMPAC package usage and modifications:

Extreme: This code, enabling the CP search for several scalar functions, was used as is but providing as input a <ρ_α -ρ_β> *wfn* file. The total density was selected as the scalar function for the CP search, but given the peculiar nature of the *wfn* file, a spin-density CP search was in reality performed.

Spinsf: This code is a modified version (C. Gatti, [19], Supplementary Information, http://www.rsc.org/suppdata/sc/c4/c4sc03988b/c4sc03988b1.pdf) of the PROMEGA/PROAIM code [42], able, *inter alias*, to evaluate the Source Function (SF) contributions at a number of reference points, given as input. The code has been further adapted by G. Bruno and G. Macetti for calculating the ∇s ZFS basins (see main text) and their local and integral SF contributions. The code adaption just involved a reading of the number and coordinates of the (3,-3) spin-density attractors, which are treated as QTAIM non-nuclear attractors by the original spinsf code. Properties of the associated basins are then obtained as a result of the integration step of the spinsf code. Clearly, the adapted spinsf code was also run using as input a <ρ_α -ρ_β> *wfn* file.

Plotden2013: This code, written by C. Gatti (19, Supplementary Information), allows for evaluating contour maps in hpgl format for many scalar functions. It was run using as input a <ρ_α -ρ_β> *wfn* file, so that the spin density and its Laplacian 2D contour maps (Figures 1-2 and S2-S5) were obtained by requiring the plot of the electron density and of its Laplacian, respectively. Analogously, spin-density differences (Figures S6-S8) were obtained from the density difference option, using the <ρ_α - ρ_β>, <ρ_α> and <ρ_β> *wfn* files for the (ρ_α - ρ_β) (Figure S6), ρ_α (Figure S7) and ρ_β (Figure S8) density differences, respectively.

Table S1. Distances, d_{ROHF}^{method} (Å), of the UHF and CAS CPs from the corresponding ROHF CPs[1].

**CP, N**	d_{ROHF}^{UHF}	d_{ROHF}^{CAS}
1	0.004	0.005
2	0.203	0.255
3	0.001	0.000
4	0.008	0.023
5	0.007	0.009
6	0.132	0.178
7	0.023	0.044
8	0.084	0.132

[1] Distances are listed for those CPs (1-8), which are common to all the three wavefunction models. CPs are labelled by their identification number N (see Tables 2-3 and Figures 1-2 of the paper.

Table S2. Lengths l (Å) of the α-α spin maxima (M1-M2) joining paths and their (3,-1) CP percentage displacements, Δ%, from the lines midpoint.

M1-M2 line	Model	l (Å)	Δ%[2]
1 – 3	ROHF	0.188	54.7
	UHF	0.194	47.3
	CAS	0.193	29.5
1 - 2	ROHF	1.607	22.0
	UHF	1.784	17.6
	CAS	1.875	14.7
1 - 1	ROHF	0.720	0
	UHF	0.703	0
	CAS	0.698	0
2 - 2	ROHF	3.298	0
	UHF	3.078	0
	CAS	3.112	0

[1] $\Delta\% = \frac{\Delta_{3,-1} \cdot 100}{0.5 \cdot l}$, where $\Delta_{3,-1}$ is the (3,-1) CP shift from the line midpoint and l is the path length. [2]The (3,-1) CP is displaced towards maximum M2 along the path (when Δ% > 0).

Table S3. Selected Properties[1] at the (3,-3) and (3,-1) critical points of the $^3B_1$ H_2O molecule for various wavefunction models.

N	M	WF	*type*	s	ρ	$\nabla^2 s$	μ_1	μ_2	μ_3
					**Maxima (3,-3)**				
1	2	ROHF	*3*	0.603	1.938	-26.978	-9.619	-9.482	-7.877
	2	UHF	*3*	0.613	1.816	-25.944	-9.375	-9.135	-7.435
	2	CAS	*3*	0.617	1.820	-25.670	-9.478	-8.915	-7.276
2	2	ROHF	*1*	0.018	0.263	-0.697	-0.264	-0.256	-0.177
	2	UHF	*2*	0.015	0.090	-0.119	-0.046	-0.037	-0.035
	2	CAS	*3*	0.012	0.072	-0.082	-0.035	-0.024	-0.023
3	1	ROHF	*1*	0.683	295.534	$-5.6*10^3$	$-1.9*10^3$	$-1.9*10^3$	$-1.8*10^3$
	1	UHF	*1*	0.840	295.432	$-6.7*10^3$	$-2.3*10^3$	$-2.2*10^3$	$-2.2*10^3$
	1	CAS	*1*	1.262	295.814	$-10.1*10^3$	$-3.6*10^3$	$-3.5*10^3$	$-3.5*10^3$
					**Saddles (3,-1)**				
4	2	ROHF	*1*	0.367	82.250	-45.534	-51.921	-48.509	54.896
	2	UHF	*1*	0.415	64.569	-47.064	-47.231	-43.491	43.658
	2	CAS	*1*	0.474	41.885	-45.172	-39.442	-36.594	30.864
5	1	ROHF	*6*	0.094	1.265	2.051	-1.691	-0.584	4.326
	1	UHF	*6*	0.130	1.308	1.206	-2.392	-0.863	4.460
	1	CAS	*6*	0.147	1.327	0.850	-2.799	-0.830	4.480
6	4	ROHF	*6*	0.006	0.085	0.023	-0.017	-0.010	0.050
	4	UHF	*6*	0.005	0.048	0.013	-0.014	-0.005	0.032
	4	CAS	*6*	0.004	0.039	0.010	-0.011	-0.004	0.025
7	1	ROHF	*3*	0.003	0.006	-0.002	-0.004	-0.001	0.002
	1	UHF	*3*	0.003	0.006	-0.003	-0.004	-0.001	0.002
	1	CAS	*3*	0.003	0.006	-0.003	-0.004	-0.001	0.002

[1] Dimensioned property in au (e^-(bohr)$^{-3}$ for electron and electron spin densities; e^-(bohr)$^{-4}$ for electron and electron-spin-density gradients; e^-(bohr)$^{-5}$ for electron and electron-spin-density Laplacians). For each CP, the CPs identification number (N), multiplicity (M) and type (see Table 1 in the main text) is reported along with the values of several properties at the CP: the electron-spin-density s, the electron density ρ, the Laplacian of the spin density $\nabla^2 s$ and the eigenvalues μ_i (i=1-3) of the Hessian matrix of $\nabla^2 s$

Table S4. Selected Properties[1] at the (3,+1) and (3,+3) Critical Points of the $^3B_1$ H_2O molecule for various wavefunction models.

N	M	WF	*type*	S	ρ	$\nabla^2 s$	μ_1	μ_2	μ_3
					**Saddles (3,+1)**				
8	2	ROHF	*6*	0.003	0.009	0.004	-0.002	0.002	0.004
	2	UHF	*6*	0.003	0.008	0.003	-0.003	0.002	0.004
	2	CAS	*6*	0.002	0.007	0.003	-0.002	0.001	0.004
9	2	ROHF	*6*	0.001	0.039	0.007	-0.001	0.001	0.008
10	1	UHF	*3*	0.000	0.0051	-0.0003	-0.0008	0.0001	0.0005
	1	CAS	*3*	0.000	0.0056	-0.0002	-0.0007	0.0001	0.0004
11	1	UHF	*6*	0.011	25.929	52.020	-0.289	20.116	32.193
	1	CAS	*6*	0.030	20.991	49.062	-0.689	21.230	28.520
12	1	UHF	*6*	-0.001	0.062	0.055	-0.004	0.018	0.041
	1	CAS	*6*	-0.002	0.063	0.059	-0.006	0.019	0.045
13	1	UHF	*6*	-0.001	0.297	0.176	-0.003	0.015	0.165
	1	CAS	*6*	-0.000	0.068	0.021	-0.000	0.005	0.016
14	2	UHF	*6*	-0.002	0.225	0.166	-0.006	0.034	0.138
	2	CAS	*6*	0.023	1.517	7.252	-0.276	0.824	6.704
					**Minima (3,-3)**				
15	2	UHF	*1*	-0.007	0.295	0.261	0.029	0.090	0.142
	2	CAS	*1*	-0.008	0.289	0.260	0.039	0.087	0.134
16	2	UHF	*2*	-0.002	0.390	0.380	0.009	0.023	0.347
	2	CAS	*3*	0.020	6.369	23.418	0.342	6.300	16.775

[1] Dimensioned property in au ($e^-(bohr)^{-3}$ for electron and electron spin densities; $e^-(bohr)^{-4}$ for electron and electron-spin-density gradients; $e^-(bohr)^{-5}$ for electron and electron-spin-density Laplacians). For the description of listed properties see footnote 1 of Table S3.

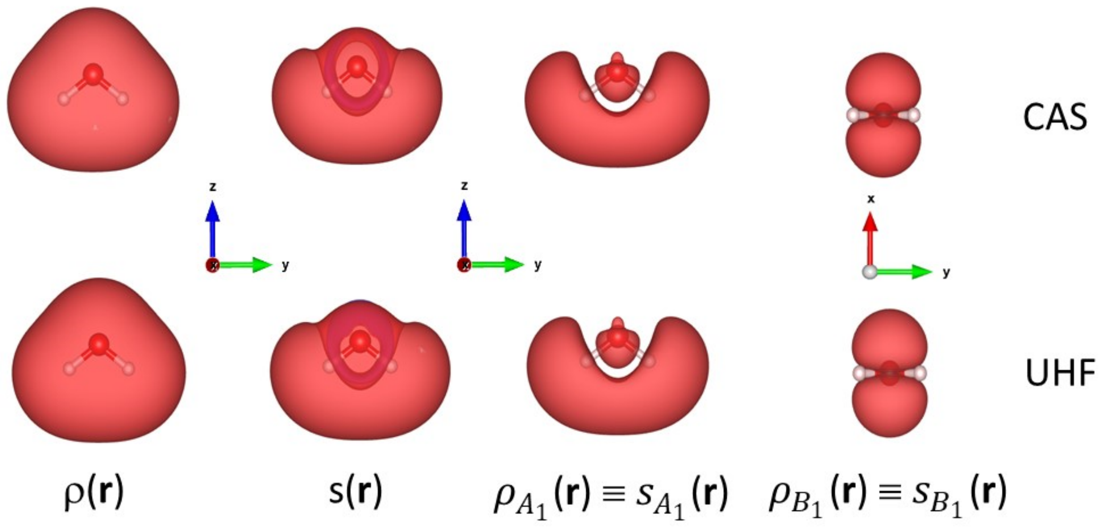

Figure S1. A 3D representation of the $^3B_1$ H_2O CAS and UHF total electron density ρ, total electron-spin-density s and electron densities associated to the two SOMOs A_1 and B_1. SOMO A_1 is dominated by O s, p_z-type functions, while SOMO B_1 is dominated by O p_x-type functions. The SOMOs A_1 and B_1 densities are equivalent to their spin densities, since SOMOs are occupied only by α-electrons. The total electron spin density assumes both positive and negative values and here are shown two s-isovalue surfaces, namely s = 0.0015 $e^-(bohr)^{-3}$ (red) and s = $-2\cdot10^{-8}$ $e^-(bohr)^{-3}$ (dark red). The 3D representations were obtained through the VESTA 3 package [44].

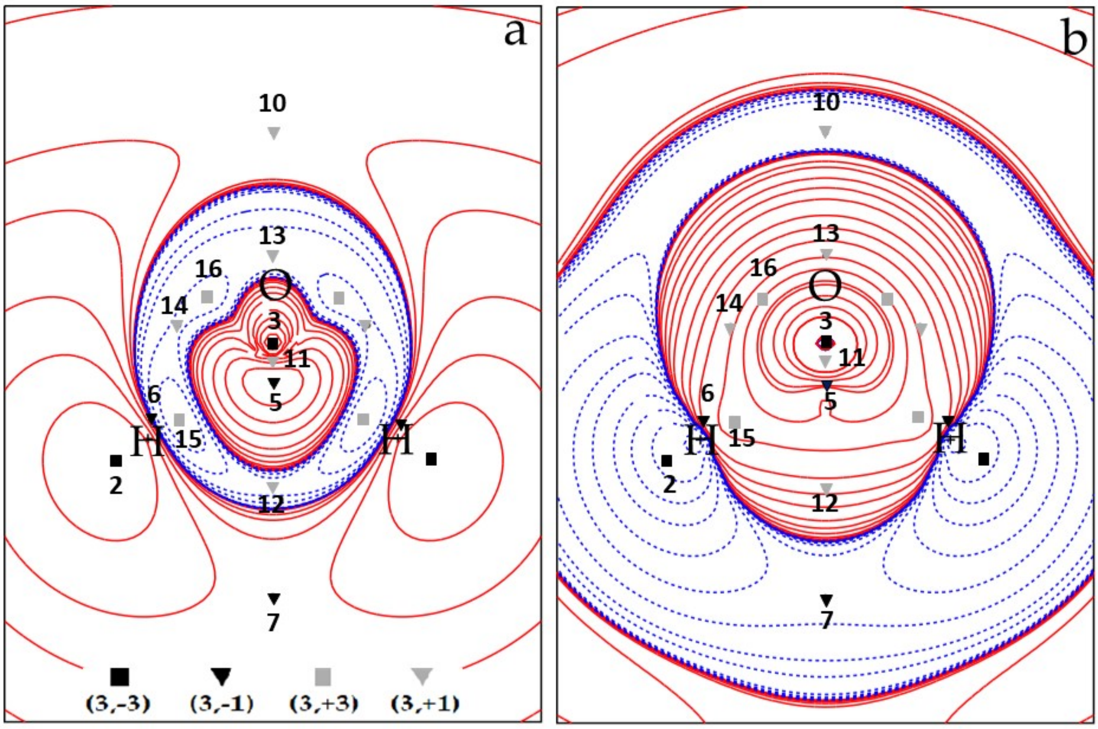

Figure S2. UHF contour plots in the σ_v plane of $^3B_1$ H_2O containing the three nuclei: (a) electron-spin-density s and (b) electron-spin-density Laplacian $\nabla^2 s$. Contour maps are drawn in intervals of ± $(2,4,8)\cdot 10^{-n}$, $0 \le n \le 6$ au ($e^-(bohr)^{-3}$ and $e^-(bohr)^{-5}$ for electron spin density and electron-spin-density Laplacian, respectively). Spin-density CPs are shown on both maps, the unique ones labelled also by their identification number N (Tables 2-3 of the main paper). CPs 6 locations are projected on this map (see text of the main paper for more details). The dashed blue contour lines correspond to negative s or $\nabla^2 s$ values. The solid squares mark (3,-3) CPs, the solid triangles (3,-1) CPs, gray triangles (3,+1) CPs and gray squares (3,+3) CPs.

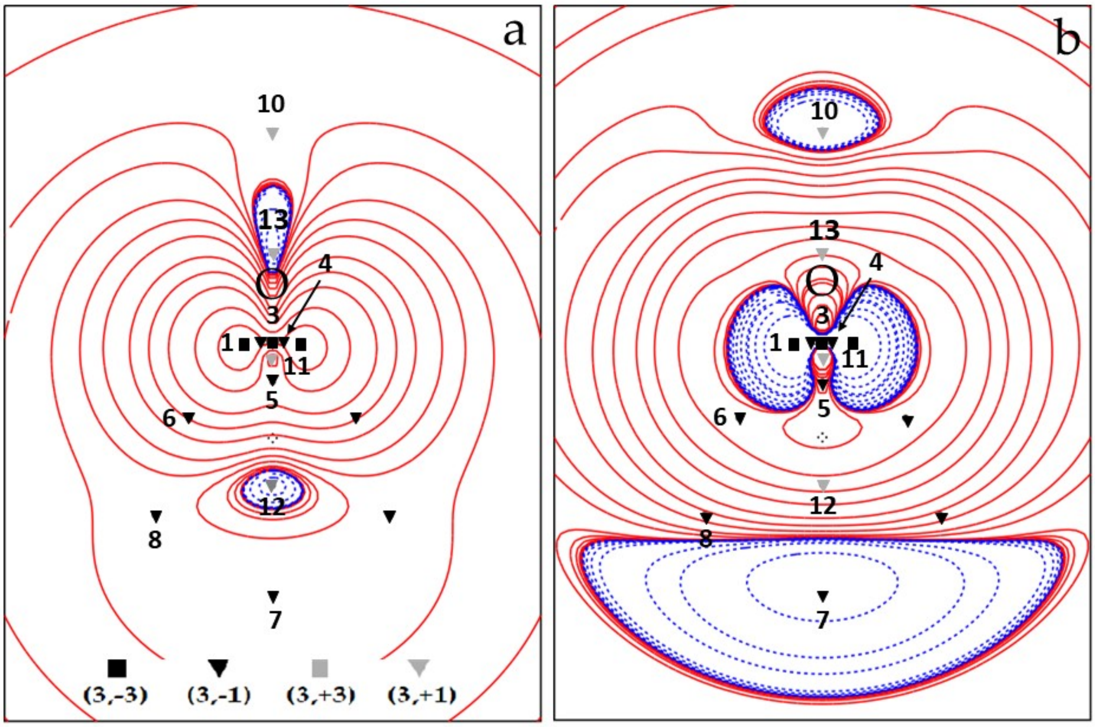

Figure S3. UHF contour plots in the $\sigma_{v'}$ plane of $^3B_1$ H_2O containing the O nucleus: (a) electron-spin-density s and (b) electron-spin-density Laplacian $\nabla^2 s$. For all other details, see the caption of Figure S2.

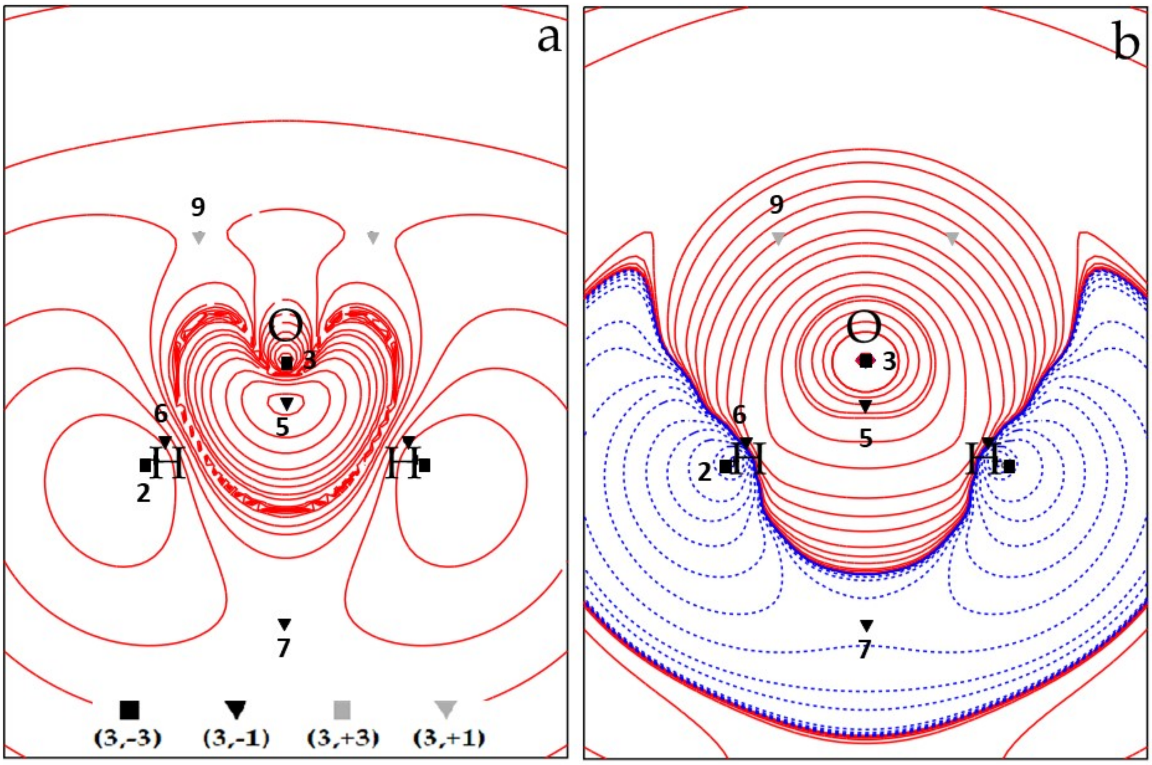

Figure S4. ROHF contour plots in the σ_v plane of $^3B_1$ H_2O containing the three nuclei: (a) electron-spin-density s and (b) electron-spin-density Laplacian $\nabla^2 s$. For all other details, see the caption of Figure S2

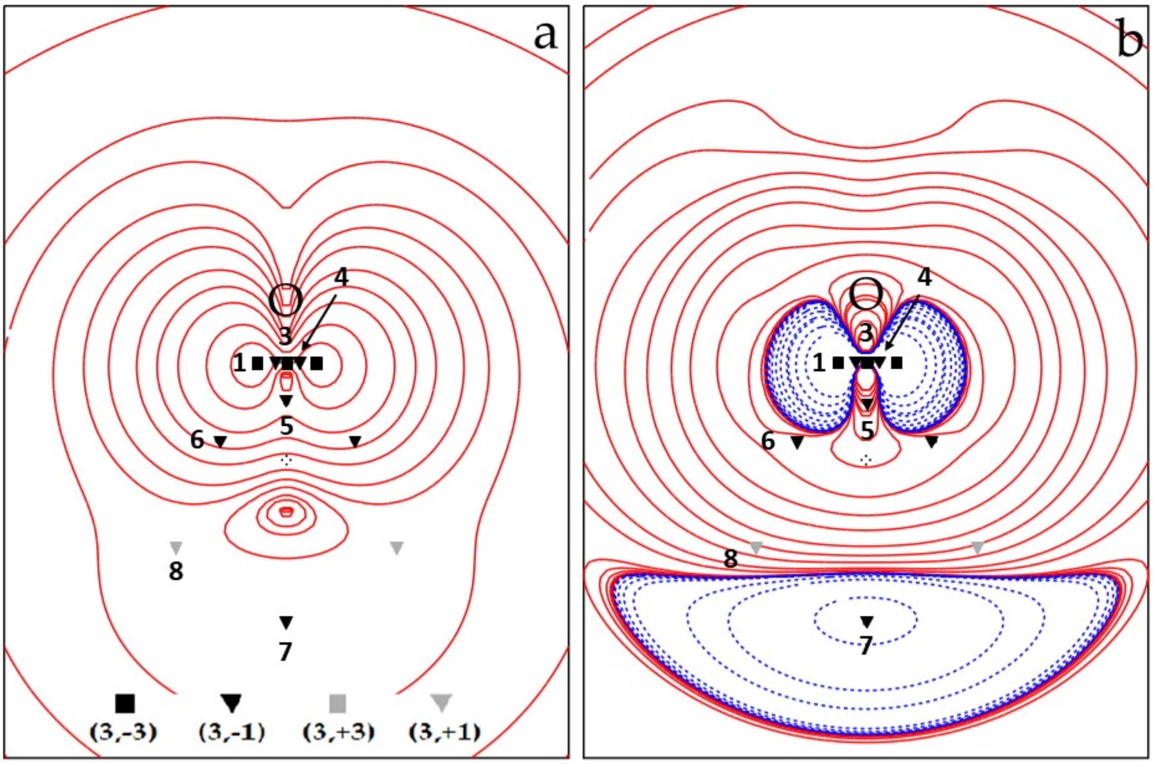

Figure S5. ROHF contour plots in the $\sigma_{v'}$ plane of $^3B_1$ H_2O containing the O nucleus: (a) electron-spin-density s and (b) electron-spin-density Laplacian ∇^2s. For all other details, see the caption of Figure S2.

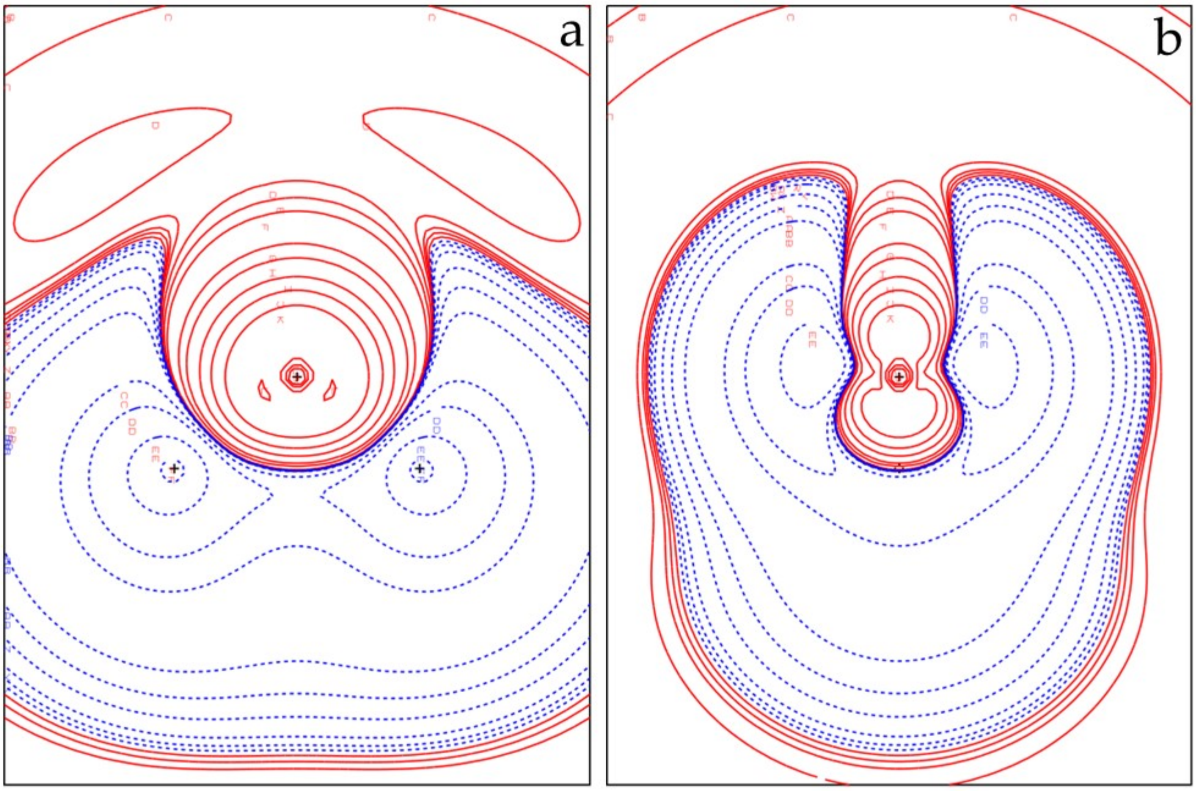

Figure S6. Spin-density difference (CAS-UHF) contour plots of the $^3B_1$ H_2O molecule in the (a) σ_v plane containing the three nuclei and (b) in the σ_v' plane containing the O nucleus. The dashed blue contour lines correspond to negative values. Contour maps are drawn in intervals of ± $(2,4,8)\cdot 10^{-n}$, $0 \le n \le 6$ au ($e^-(bohr)^{-3}$ and $e^-(bohr)^{-5}$ for electron spin density and electron-spin-density Laplacian, respectively).

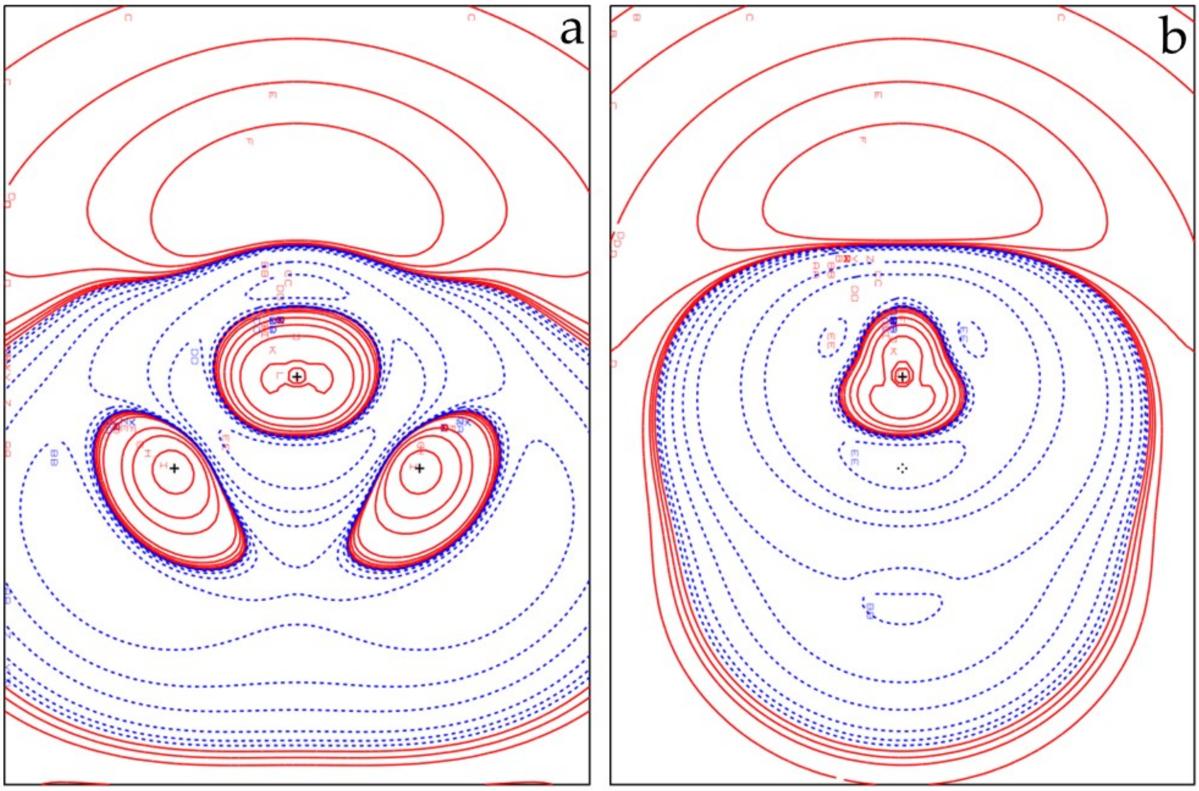

Figure S7. ρ_α density difference (CAS-UHF) contour plots of the $^3B_1$ H_2O molecule in the (a) σ_v plane containing the three nuclei and (b) in the $\sigma_{v'}$ plane containing the O nucleus. For all other details, see the caption of Figure S6

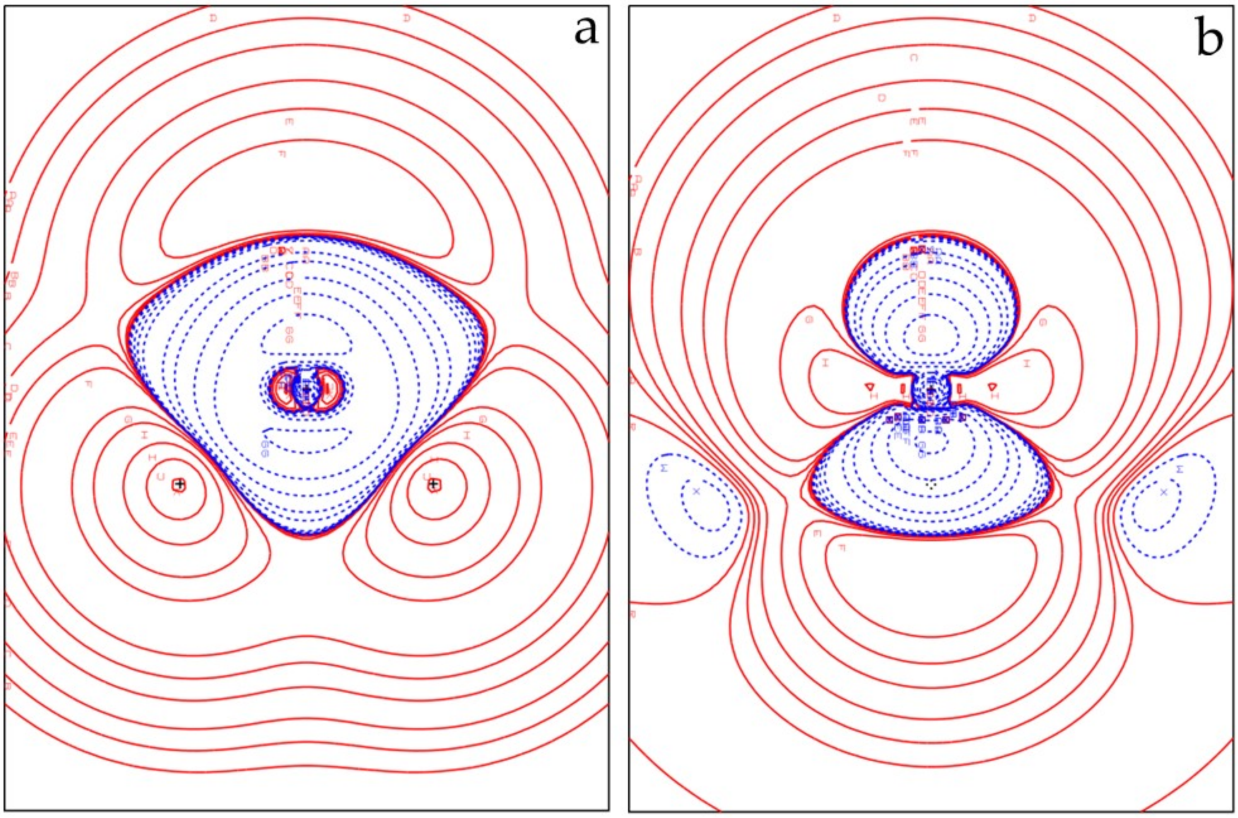

Figure S8. ρ_β density difference (CAS-UHF) contour plots of the $^3B_1$ H_2O molecule in the (a) σ_v plane containing the three nuclei and (b) in the σ_v' plane containing the O nucleus. For all other details, see the caption of Figure S6

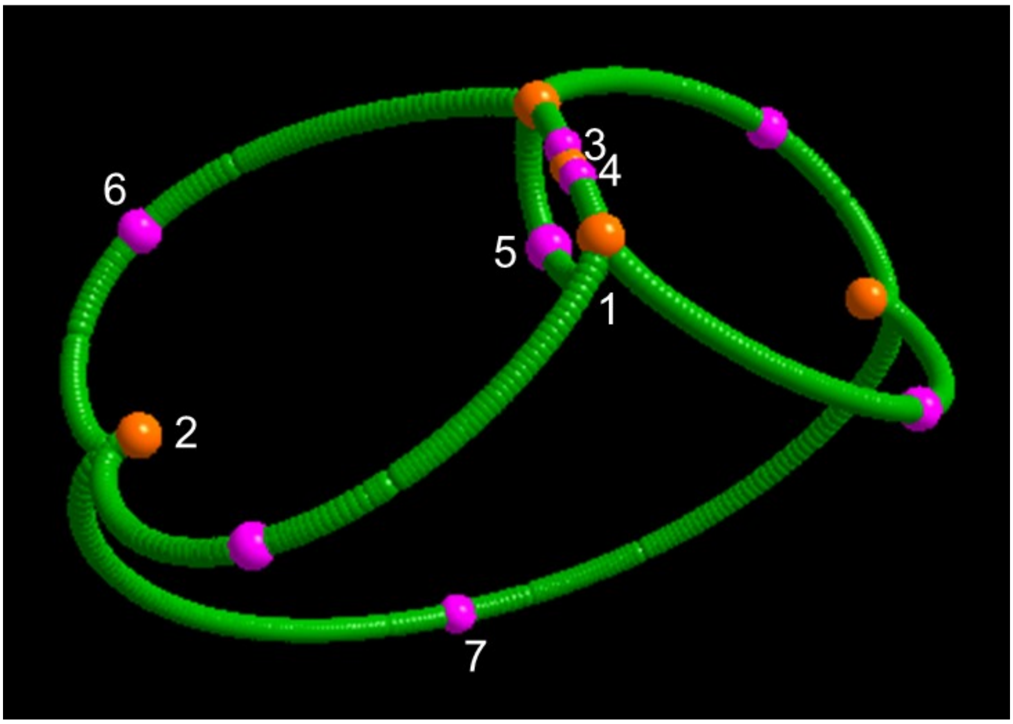

Figure S9. $^3B_1$ H_2O CAS (α-α) molecular magnetic spin graph. Critical points of s_{mag} are labelled as in Tables 2 and 3 (main text) and portrayed as colored balls [(3,-3) s maxima, orange; (3,-1) s saddles, violet; (3,+3) s minima or –s maxima, blue; (3,+1) s ring or –s (3,-1) saddles, yellow]. The spin maxima joining paths are represented as thick green wires. The picture was obtained through the code Diamond v3.21 [38]
